# Risk of Cardiovascular Disease Due to General Anesthesia and Neuraxial Anesthesia in Lower-Limb Fracture Patients: A Retrospective Population-Based Cohort Study

**DOI:** 10.3390/ijerph17010033

**Published:** 2019-12-18

**Authors:** Han-Wei Yeh, Liang-Tsai Yeh, Ying-Hsiang Chou, Shun-Fa Yang, Sai-Wai Ho, Ying-Tung Yeh, Ying-Ting Yeh, Yu-Hsun Wang, Chi-Ho Chan, Chao-Bin Yeh

**Affiliations:** 1 School of Medicine, Chang Gung University, Taoyuan City 333, Taiwan; george66889@gmail.com; 2 Institute of Medicine, Chung Shan Medical University, Taichung 402, Taiwan; 68990@cch.org.tw (L.-T.Y.); hideka.chou@gmail.com (Y.-H.C.); ysf@csmu.edu.tw (S.-F.Y.); 3 Department of Anesthesiology, Changhua Christian Hospital, Changhua 500, Taiwan; 4 Department of Medical Imaging and Radiological Sciences, Chung Shan Medical University, Taichung 402, Taiwan; 5 Department of Radiation Oncology, Chung Shan Medical University Hospital, Taichung 402, Taiwan; 6 Department of Medical Research, Chung Shan Medical University Hospital, Taichung 402, Taiwan; cshe731@csh.org.tw; 7 Department of Emergency Medicine, School of Medicine, Chung Shan Medical University, Taichung 402, Taiwan; hswk@ymail.com; 8 Department of Emergency Medicine, Chung Shan Medical University Hospital, Taichung 402, Taiwan; 9 School of Dentistry, Chung Shan Medical University, Taichung 402, Taiwan; yehtungtung@hotmail.com (Y.-T.Y.); candyyeh8989@gmail.com (Y.-T.Y.); 10 Department of Dentistry, Chung Shan Medical University Hospital, Taichung 402, Taiwan; 11 Department of Microbiology and Immunology, Chung Shan Medical University, Taichung 402, Taiwan

**Keywords:** cardiovascular diseases, general anesthesia, neuraxial anesthesia

## Abstract

The purpose of this study was to analyze the relationship between elevated cardiovascular disease (CVD) risk and type of anesthesia by using the National Health Insurance Research Database (NHIRD) of Taiwan in a one-year follow-up period. We assessed whether general anesthesia (GA) or neuraxial anesthesia (NA) increased CVD occurrence in lower-limb fracture patients. Approximately 1 million patients were randomly sampled from the NHIRD registry. We identified and enrolled 3437 lower-limb fracture patients who had received anesthesia during operations conducted in the period from 2010 to 2012. Next, patients were divided into two groups, namely GA (n = 1504) and NA (n = 1933), based on the anesthetic technique received during surgery. Our results revealed that those receiving GA did not differ in their risk of CVD relative to those receiving NA, adjusted HR = 1.24 (95% CI: 0.80–1.92). Patients who received GA for more than 2 h also did not differ in their risk of CVD relative to those receiving NA for less than 2 h, adjusted HR = 1.43 (95% CI: 0.81–2.50). Moreover, in the GA group (i.e., patients aged ≥65 years and women), no significant difference for the risk of CVD events was observed. In conclusion, in our study, the difference in the risk of CVD between lower-limb fracture patients receiving NA and GA was not statistically significant. The incidence rate of CVD seemed to be more correlated with patients’ underlying characteristics such as old age, comorbidities, or admission to the intensive care unit. Due to the limited sample size in this study, a database which reviews a whole national population will be required to verify our results in the future.

## 1. Introduction

Anesthetic techniques can be classified as general anesthesia (GA), regional (caudal and neuraxial (spinal and epidural)) anesthesia, and peripheral nerve block. Choosing a suitable anesthetic technique can have benefits for patients. For some patients, physicians need to determine whether GA or neuraxial anesthesia (NA) is more suitable for a particular surgical procedure. GA has been reported to be a risk factor for dementia. In a mouse experiment, anesthetizing mice with ether vapors or sodium pentobarbital for short periods (30 s to 5 min) induced tau phosphorylation. In humans, this might increase the risk of postoperative cognitive impairment and the risk for Alzheimer’s disease. [1]. Isoflurane-treated AbetaPP mice had increased beta-amyloid protein aggregates in the brain [2]. Studies on the relationship between GA and AD have yielded inconsistent results. Another example includes a primiparous female who underwent cesarean delivery with epidural anesthesia, but not with spinal anesthesia, who was suggested to have an increased risk of subsequent chronic low back pain [3].

Cardiovascular disease (CVD) is one of the most common causes of morbidity and mortality worldwide. To date, CVD is the second leading cause of death in Taiwan. According to the definition of the World Health Organization, CVD is a group of disorders of the heart and blood vessels including coronary heart disease, cerebrovascular disease, peripheral arterial disease, rheumatic heart disease, congenital heart disease, deep vein thrombosis, and pulmonary embolism. Risk factors for CVD have been divided into invariable and variable risk factors. Invariable risk factors include age, sex, and genetic status [4]. However, known variable risk factors include improper lifestyle habits, such as smoking tobacco and poor exercise and eating habits. Other risk factors associated with CVD include the presence of underlying diseases such as arterial hypertension, diabetes mellitus, dyslipidemia, cholesterol, and obesity. In addition, myocardial injury after noncardiac surgery (MINS) might be important factor in cardiovascular events during perioperative complications [5]. Hip fracture was found to be one of the risk factors for MINS during the pre-operative period [6].

Anesthesia types are unique to each individual patients with ASA (American Society of Anesthesiologists) physical status or multiple comorbidities and types of surgery, even in different hospital [7]. As such, the purpose of physicians and anesthesiologists in deciding the method of anesthesia (GA or NA) is to reduce the risk of complications, promote faster recovery, minimize surgical stress response, and improve post anesthesia outcome [8]. In previous literature reviews, the advantages and risks of GA and NA have been compared. For cesarean section, NA was linked with a shorter duration of hospitalization than GA [9]. The opioid-sparing effects of NA were associated with a better quality of recovery in patients after the surgical procedure [10]. A meta-analysis indicated that the use of NA for hip joint replacement may have better outcomes, including a lower blood transfusion requirement and a decrease in deep venous thrombosis or pulmonary embolism and serious complications [11]. In addition, the long-term effects of anesthesia remain unknown. Although NA may be associated with a relatively lower risk on several aspects and a better outcome when compared with GA, more studies are required for verification. Therefore, to determine whether GA or NA have shown benefits and better outcome for patient with lower limbs fracture surgery, we compared the association of GA and NA with CVD risk in lower-limb fracture patients at the one-year follow-up period. The purpose of this study was to investigate the risk of CVD in patients with GA and NA. The hypothesis was that GA and NA have significant differences in CVD risk.

## 2. Experimental Section

### 2.1. Data Sources

The Longitudinal Health Insurance Database (LHID) is managed by the Taiwan National Health Research Institutes. One million randomly sampled beneficiaries were recruited with all outpatient and inpatient medical claims including drug medications, medical operations, procedures, and fees. The study was approved by the Ethical Review Board of Chung Shan Medical University Hospital (CS18096).

### 2.2. Study Groups

This study used a retrospective cohort study design. All recruited patients were aged 18 years or older, had lower-limb fractures (ICD-9-CM codes 820–827), and underwent an operation with anesthesia between 1 January 2010, and 31 December 2012. The index date was defined as the first date of lower-limb fracture. To eliminate confounding from a past disease or re-anesthesia surgery, we excluded patients with a CVD diagnosis before the index date or the requirement of anesthesia surgery again during the study period. The study population was divided into GA and NA groups by surgical codes (96005C, 96006C, 96007C, 96008C, 96017C, 96018C, 96019C, 96020C, 96021C, and 96022C). NA included spinal and epidural anesthesia.

### 2.3. Outcome and Covariate Measurement

The study endpoint was the diagnosis of new-onset CVD, including ischemic heart disease (ICD-9-CM codes 410, 411, 413, 414), heart failure (ICD-9-CM codes 428), and cerebrovascular disease (ICD-9-CM codes 430–437) during emergency or hospitalization. All patients were followed until one year until the occurrence of CVD, withdrawal from the social insurance system, or the end of follow-up, whichever came first. Covariates were age, sex, hypertension (ICD-9-CM codes 401–405), hyperlipidemia (ICD-9-CM codes 272.0–272.4), diabetes (ICD-9-CM code 250), renal disease (ICD-9-CM codes 582, 583.0–583.7, 585, 586, and 588), liver disease (ICD-9-CM codes 456.0, 456.1, 456.2, 571.2, 571.4, 571.5, 571.6, 572.2, 572.3, 572.4, and 572.8), chronic pulmonary disease (ICD-9-CM codes 490–496, 500–505, and 506.4), and usage of anticoagulants (aspirin/clopidogrel/warfarin). From the LHID, we were unable to obtain personal behavioral information such as smoking, alcohol consumption, and laboratory data. In this study, we used chronic pulmonary disease and liver disease as substitutes for the indirect control of smoking and alcohol consumption interference. Other comorbidity choices, such as hypertension, hyperlipidemia, diabetes, and renal disease are all potential CVD risk factors. These covariates were defined before the index date. Furthermore, admission into the intensive care unit (ICU) was considered to control the severity of lower-limb fractures in both groups.

### 2.4. Statistical Analysis

To compare GA and NA, the chi-squared test or independent t test was used, as appropriate. The cumulative incidence of CVD cases was analyzed using Kaplan–Meier analysis, and the significance was calculated using the log-rank test. The Cox proportional hazard model was used to estimate the hazard ratios of CVDs. A sensitivity analysis of risk of CVD events was also conducted for different follow-up periods. Statistical analyses were performed using SPSS V.18.0 (SPSS, Chicago, Il, USA). A *p*-value less than 0.05 was considered statistically significant.

## 3. Results

### 3.1. Characteristics of Study Subjects

**Figure 1:**
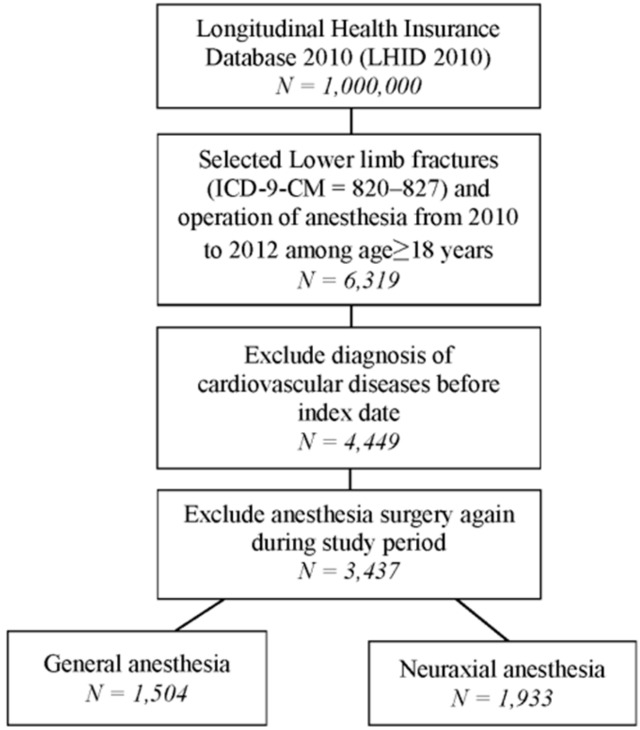
Flow chart for patient selection.

A total of 6319 patients with lower-limb fractures who received anesthesia during an operation were identified from 2010 to 2012. After excluding patients with CVD diagnosis and repeated anesthesia surgery during the study period, 3437 patients were enrolled in the study. Furthermore, patients were divided into two groups. One group received GA (n = 1504) and the other group received NA (n = 1933) during the surgery for lower-limb fractures (Figure 1). The distributions of age, sex, comorbidities, ICU admission, anticoagulants, anesthesia time, and hospital levels among patients receiving GA and NA are presented in Table 1. The mean age and standard deviation of patients receiving GA and NA were 50.7 ± 20.8 and 57.6 ± 20.8 years, respectively. The proportions of male patients receiving GA and NA were 52.1% and 50.7%, respectively.

### 3.2. Risk of CVD with General Anesthesia, Neuraxial Anesthesia, Age, Gender, Different Comorbidities, and Other Conditions

**Figure 2:**
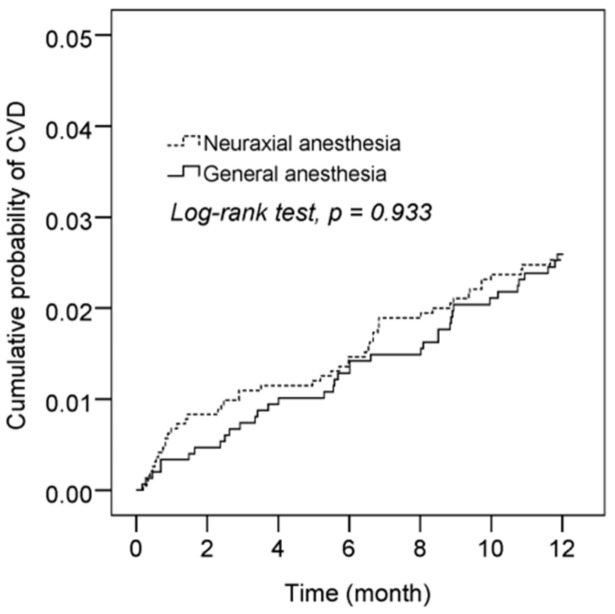
Kaplan–Meier curves with a cumulative probability of cardiovascular disease (CVD) following GA and NA in lower-limb fracture patients.

We estimated the cumulative incidence of CVD cases in patients who received GA and NA. The Kaplan–Meier curve of the cumulative probability of CVDs indicated that the GA group did not have a higher risk of CVDs within 1 year (log-rank test, *p* = 0.933; Figure 2). The Cox proportional hazard model revealed that the incidence of CVD did not differ in patients receiving GA than in patients receiving NA, HR = 1.24 (95% CI: 0.80–1.92). After adjustment for age, sex, comorbidities, ICU admission, and anticoagulants, patients aged 65 years or older (adjusted hazard ratio (HR): 5.77; 95% confidence interval [CI]: 3.21–10.38), male patients (adjusted HR: 1.89; 95% CI: 1.21–2.95), and patients with comorbidities, such as diabetes (adjusted HR: 2.09; 95% CI: 1.29–3.37), had a higher risk of developing a CVD. Patients who had been admitted to the ICU due to lower-limb fractures also had a higher risk of a CVD event occurring (adjusted HR: 2.59; 95% CI: 1.35–4.96; Table 2).

### 3.3. Risk of CVD Between General Anesthesia and Neuraxial Anesthesia Patients and Specific Subgroup Characteristics

To determine whether the duration of both GA and NA affects the risk of CVD, both anesthesia groups were divided into less than 2 h and more than 2 h groups. Compared with patients who received NA for less than 2 h, patients who received GA for more than 2 h did not differ in their risk of developing a CVD (adjusted HR: 1.43; 95% CI: 0.81–2.50; Table 3). We also conducted a subgroup analysis between GA and NA patients. In stratification of age and gender, those receiving GA did not differ in their risk of CVD relative to those receiving NA (Table 4). A sensitivity analysis was conducted, which showed that the GA and NA groups did not have statistically significant differences in different follow-up periods (Table 5).

## 4. Discussion

In this study, a total of 3437 lower-limb fracture patients who received GA (n = 1504) or NA (n = 1933) were enrolled. The risk of CVD events did not differ in general anesthesia when compared with neuraxial anesthesia in the Cox proportional hazard model. To date, no studies have reported either GA or NA as a risk factor for the development of CVD. In contrast, CVDs have been described as postoperative outcomes of any type of anesthesia [12,13,14]. Generally, the postoperative time frame can range from 24 h to 30 days [13]. In our study, the average onset of CVDs in patients who received NA and GA was 4.8 and 6.0 months, respectively (Appendix A Table A1).

Risk factors for CVDs have accumulated rapidly. Underlying diseases, such as hypertension, atrial fibrillation, kidney failure, diabetes, aging, and sex are also risk factors for CVDs that have been reported for more than 20 years [4,15]. In our study, patients aged ≥65 years, male patients, and patients with diabetes were shown to have a higher risk of developing a CVD. 

In this study, we found that lower-limb fracture patients admitted to the ICU had a higher risk of developing CVD relative to those lower-limb fracture patients that underwent surgery but through direct hospital admission. In fact, several studies have indicated that VTE might be a predisposing factor of CVDs [16,17,18]. In fact, patients with particularly serious diseases staying in the ICU could also have an increased risk of CVD. A cohort study indicated that intensive care patients with acute kidney injury (AKI) had an increased risk of heart failure or myocardial infarction up to three years after hospital discharge [19].

Recently, two meta-analyses indicated an association between intra-operative hypotension (IOH) and MINS. Gu’s group reviewed 14 cohort studies of IOH and MINS. They found that IOH was associated with increased risks of 30-day mortality and major adverse cardiac events (MACEs), especially myocardial injury and AKI after non-cardiac surgery [20]. Similarly, An’s group reviewed 15 observational studies of IOH and MINS. They found that IOH was associated with an increased risk of postoperative AKI and myocardial injury. In more detail, the duration of IOH lasting only more than 5 min was associated with an increased risk of 30-day mortality [21]. In particular, patients suffering from pre-existing CVDs may have increased risks of perioperative cardiovascular morbidity and mortality following noncardiac surgery. Therefore, physicians and anesthesiologists need to consider cardioprotective medication, anesthetic regimen choice, blood pressure management, and transfusion regimens carefully in order to decrease such CVD risk [22]. The development of intraoperative hypotension is known to be associated with MINS, intraoperative vasopressor use, postoperative sepsis, number of ventilator days, and the types and quantities of ICU sedative and narcotic use, just to name a few contributors.

The current study has some limitations. First, we did not have access to potentially relevant personal behavioral information such as smoking habits, alcohol consumption, body mass index, and the severity of lower-limb fracture. This is significant because smoking, drinking, and BMI (obesity) are risk factors for CVD, and these factors may be diversifying the type of anesthesia. Second, the dataset did not provide information regarding the types of anesthetic technique used in patients, clinical presentations, or laboratory data. Patients’ individual statuses, presence of severity of lower limbs fracture and bleeding profile laboratory data may determine the type of anesthesia method selected by anesthesiologist. Third, the study subjects were not selected from the national population. Therefore, in consequence of limiting by the sample sizes that the study results may exist a wide confidence interval and corresponding lower power.

## 5. Conclusions

In summary, our study indicates that the difference in the risk of CVD between lower-limb fracture patients receiving NA and GA was not statistically significant. Owing to the limited sample size, further experiments are required to identify whether different anesthetic procedures are associated with differential risks of CVD in the future. For the application of anesthesia in different diseases, further study is needed for verification. Therefore, physicians themselves might decide to use GA or NA under different conditions.

## Figures and Tables

**Table 1:** Demographic characteristics of general anesthesia (GA) and neuraxial anesthesia (NA) groups.

Variables	General Anesthesia (N = 1504)		Neuraxial Anesthesia (N = 1933)		*p*-Value
	N	%	N	%	
Age					<0.001
<65	1080	71.8	1176	60.8	
≥65	424	28.2	757	39.2	
Mean ± SD	50.7 ± 20.8		57.6 ± 20.8		<0.001
Gender					0.406
Female	720	47.9	953	49.3	
Male	784	52.1	980	50.7	
Hypertension	384	25.5	565	29.2	0.016
Hyperlipidemia	107	7.1	200	10.3	0.001
Diabetes	204	13.6	321	16.6	0.014
Renal disease	39	2.6	56	2.9	0.590
Liver disease	80	5.3	71	3.7	0.019
Chronic pulmonary disease	88	5.9	153	7.9	0.019
Intensive care unit (ICU) admission	127	8.4	44	2.3	<0.001
Anticoagulants	69	4.6	107	5.5	0.211
Anesthesia time					<0.001
<2 h	742	49.3	1377	71.2	
≥2 h	762	50.7	556	28.8	
Hospital level					<0.001
Medical centers	553	36.8	480	24.8	
Regional hospitals	725	48.2	826	42.7	
District hospitals	180	12.0	599	31.0	
Clinics	46	3.1	28	1.4	

**Table 2:** Cox proportional hazard model of risk of CVD events among GA and NA groups.

Variables	No. of CVD Events	Observed Person-Years	Incidence Density (Per 1000 Person-Years)	Crude HR	95% CI	*p*-Value	Adjusted HR ^†^	95% CI	*p*-Value
Group									
Neuraxial anesthesia	48	1861	25.8	1			1		
General anesthesia	38	1447	26.3	1.02	0.67–1.56	0.933	1.24	0.80–1.92	0.334
Age									
<65	18	2221	8.1	1			1		
≥65	68	1087	62.6	7.67	4.56–12.90	<0.001	5.77	3.21–10.38	<0.001
Gender									
Female	43	1616	26.6	1			1		
Male	43	1692	25.4	0.95	0.63–1.46	0.829	1.89	1.21–2.95	0.005
Hypertension	52	886	58.7	4.16	2.70–6.42	<0.001	1.63	0.98–2.70	0.059
Hyperlipidemia	14	293	47.8	2.00	1.13–3.54	0.018	0.91	0.49–1.66	0.749
Diabetes	35	485	72.2	3.97	2.58–6.11	<0.001	2.09	1.29–3.37	0.003
Renal disease	8	78	102.7	4.18	2.02–8.66	<0.001	1.48	0.70–3.12	0.305
Liver disease	3	142	21.1	0.80	0.25–2.54	0.707	0.58	0.18–1.84	0.354
Chronic pulmonary disease	11	221	49.8	2.04	1.08–3.84	0.027	1.21	0.64–2.30	0.561
ICU admission	11	142	77.3	3.22	1.71–6.06	<0.001	2.59	1.35–4.96	0.004
Anticoagulants	14	159	87.8	3.81	2.15–6.75	<0.001	1.80	0.97–3.31	0.061

CVD: cardiovascular disease. ^†^ Adjusted for age, gender, hypertension, hyperlipidemia, diabetes, renal disease, liver disease, chronic pulmonary disease, ICU admission, and anticoagulant use.

**Table 3:** Subgroup analysis of the Cox proportional hazard model at anesthesia time.

Variables	No. of CVD Events	Observed Person-Years	Incidence Density (Per 1000 Person-Years)	Crude HR	95% CI	*p*-Value	Adjusted HR ^†^	95% CI	*p*-Value
Group									
NA < 2 h	32	1326	24.1	1			1		
NA ≥ 2 h	16	535	29.9	1.24	0.68–2.26	0.482	1.00	0.55–1.83	0.999
GA < 2 h	15	720	20.8	0.86	0.47–1.60	0.641	1.05	0.57–1.95	0.866
GA ≥ 2 h	23	727	31.7	1.31	0.77–2.24	0.322	1.43	0.81–2.50	0.217

CVD: cardiovascular disease. ^†^ NA (neuraxial anesthesia) and GA (general anesthesia). Adjusted for age, gender, hypertension, hyperlipidemia, diabetes, renal disease, liver disease, chronic pulmonary disease, ICU admission, and anticoagulant use.

**Table 4:** Subgroup analysis of Cox proportional hazard model using age and sex.

	General Anesthesia		Neuraxial Anesthesia		HR	95% CI	*p*-Value
	N	No. of CVD Events	N	No. of CVD Event			
Age ^†^							
<65	1080	7	1176	11	0.60	0.21–1.66	0.323
≥65	424	31	757	37	1.49	0.92–2.43	0.107
sex ^‡^							
Female	720	22	953	21	1.68	0.92–3.07	0.093
Male	784	16	980	27	0.84	0.43–1.66	0.619

CVD: cardiovascular disease. ^†^ Adjusted for sex, hypertension, hyperlipidemia, diabetes, renal disease, liver disease, chronic pulmonary disease, ICU admission, and anticoagulant use. ^‡^ Adjusted for age, hypertension, hyperlipidemia, diabetes, renal disease, liver disease, chronic pulmonary disease, ICU admission, and anticoagulant use.

**Table 5:** Sensitivity analysis of risk of CVD events in different follow-up periods.

	N	No. of CVD Event	Adjusted HR ^†^	95% CI	*p*-Value
Follow-up period ≤6 months					
Group					
Neuraxial anesthesia	1933	28	1		
General anesthesia	1504	19	1.05	0.58–1.91	0.875
Follow-up period 7–12 months					
Group					
Neuraxial anesthesia	1859	20	1		
General anesthesia	1444	19	1.52	0.79–2.91	0.206

CVD: cardiovascular disease. ^†^ Adjusted for age, sex, hypertension, hyperlipidemia, diabetes, renal disease, liver disease, chronic pulmonary disease, ICU admission, and anticoagulant use.

## Appendix A

**Table A1:** Track time of CVD following general anesthesia and neuraxial anesthesia.

	Neuraxial Anesthesia	General Anesthesia
Follow-up duration (month, mean ± SD)	11.6 ± 2	11.5 ± 2.0
Time to CVD (month, mean ± SD), N = 86	4.8 ± 3.6	6.0 ± 3.8

CVD: cardiovascular diseases.
